# Rapid Regulation of Human Multidrug and Extrusion Transporters hMATE1 and hMATE2K

**DOI:** 10.3390/ijms21145157

**Published:** 2020-07-21

**Authors:** Marta Kantauskaitė, Anna Hucke, Moritz Reike, Sara Ahmed Eltayeb, Chuyan Xiao, Vivien Barz, Giuliano Ciarimboli

**Affiliations:** Medicine Clinic D, Experimental Nephrology, University Hospital of Münster, 48149 Münster, Germany; marcikee@gmail.com (M.K.); anna_hucke@gmx.de (A.H.); moritz.reike@gmx.de (M.R.); Sara.Eltayeb@ukmuenster.de (S.A.E.); chuyanxiao_deutsch@163.com (C.X.); Vivien.Barz@ukmuenster.de (V.B.)

**Keywords:** organic cations, transport, kidneys, regulation

## Abstract

Vectorial transport of organic cations (OCs) in renal proximal tubules is mediated by sequential action of human OC transporter 2 (hOCT2) and human multidrug and toxic extrusion protein 1 and 2K (hMATE1 and hMATE2K), expressed in the basolateral (hOCT2) and luminal (hMATE1 and hMATE2K) plasma membranes, respectively. It is well known that hOCT2 activity is subjected to rapid regulation by several signaling pathways, suggesting that renal OC secretion may be acutely adapted to physiological requirements. Therefore, in this work, the acute regulation of hMATEs stably expressed in human embryonic kidney cells was characterized using the fluorescent substrate 4-(4-(dimethylamino)styryl)-N-methylpyridinium (ASP^+^) as a marker. A specific regulation of ASP^+^ transport by hMATE1 and hMATE2K measured in uptake and efflux configurations was observed. In the example of hMATE1 efflux reduction by inhibition of casein kinase II, it was also shown that this regulation is able to modify transcellular transport of ASP^+^ in Madin–Darby canine kidney II cells expressing hOCT2 and hMATE1 on the basolateral and apical membrane domains, respectively. The activity of hMATEs can be rapidly regulated by some intracellular pathways, which sometimes are common to those found for hOCTs. Interference with these pathways may be important to regulate renal secretion of OCs.

## 1. Introduction

Kidneys are key players in the excretion of several substances of endogenous and exogenous origin. Besides by filtration in glomeruli, renal excretory function is sustained by secretion through the proximal tubules [1,2]. Tubular secretion is especially important for polar and charged molecules, which are subjected to a vectorial transport across proximal tubule cells by the orchestrated action of membrane transporters expressed either in the basolateral (facing blood) or apical (facing primary urine) membrane of the cells. In vectorial secretion processes, transporters expressed in the basolateral plasma membrane domain are responsible for the influx, whereas the ones expressed in the apical membrane domain are responsible for the efflux of substrates [3]. Tubular secretion is responsible for renal excretion of charged molecules, such as organic cations (OCs). Endogenous OCs are substances with important physiological function, such as serotonin and histamine, or are metabolism products, such as creatinine. A vast majority of exogenous OCs is represented by drugs such as metformin, verapamil, morphine, etc. [4,5].

**Figure 1:**
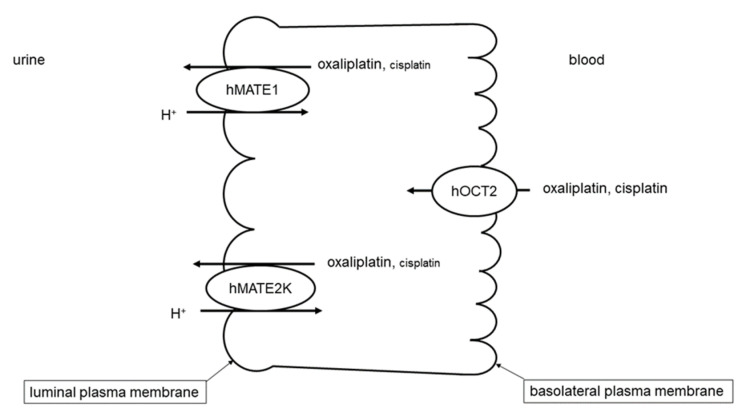
Renal secretion system for organic cations (OCs). In renal proximal tubule cells, the uptake of OCs from the blood is mediated by human organic cation transporter 2 (hOCT2) expressed on the basolateral domain of plasma membrane. The OC secretion from the cells into the urine is mediated by human multidrug and toxic extrusion protein 1 and 2K (hMATE1 and hMATE2K) expressed on the apical domain of plasma membrane. The slightly acidic pH of the urine favors secretion driven by hMATEs. In this example, oxaliplatin and cisplatin as substrates of the renal secretion system are shown. Lower affinity of cisplatin for hMATEs is indicated by the smaller font size.

The human organic cation transporter 2 (hOCT2) expressed in the basolateral membrane of proximal tubule cells mediates the first step of OC secretion, that is, the Na^+^- and H^+^-independent uptake of OCs [6,7] from the blood. The OC secretion into the urine is then accomplished by extrusion into the tubule lumen, a process that in humans is mediated mainly by human multidrug and toxin extrusion proteins 1 and 2K (hMATE1-2K) [8], pH-dependent transporters expressed in the apical membrane domain of proximal tubule cells [9,10,11,12,13]. This process is stimulated by the slightly acidic pH of primary urine. Substances that interact with hOCT2 can also interact with hMATEs [14,15,16], confirming that these transporters constitute a secretory axis for OCs in renal tubules. This transport vectorial system is involved also in the renal secretion of atypical substrates such as the chemotherapeutic drugs cisplatin and oxaliplatin. Since oxaliplatin is a better substrate of hMATE1 and hMATE2K than cisplatin, it is efficiently eliminated into the urine and causes less nephrotoxicity than cisplatin (Figure 1) [12,17,18].

It is well known that the activity of OCTs is subjected to specific acute regulation by several signaling pathways, which can elicit changes in transporter affinity or alter the number of transporters in the membrane [19]. However, information on regulation of MATEs is limited. The knowledge of specific regulation of transporters belonging to the same functional axis such as OCTs/MATEs is important, because such a regulation has the potential to modify the renal OC secretion, in this way changing body exposition to drugs and extent of nephrotoxicity. Therefore, in this work, the acute regulation of hMATEs has been comparatively investigated.

## 2. Results

**Figure 2:**
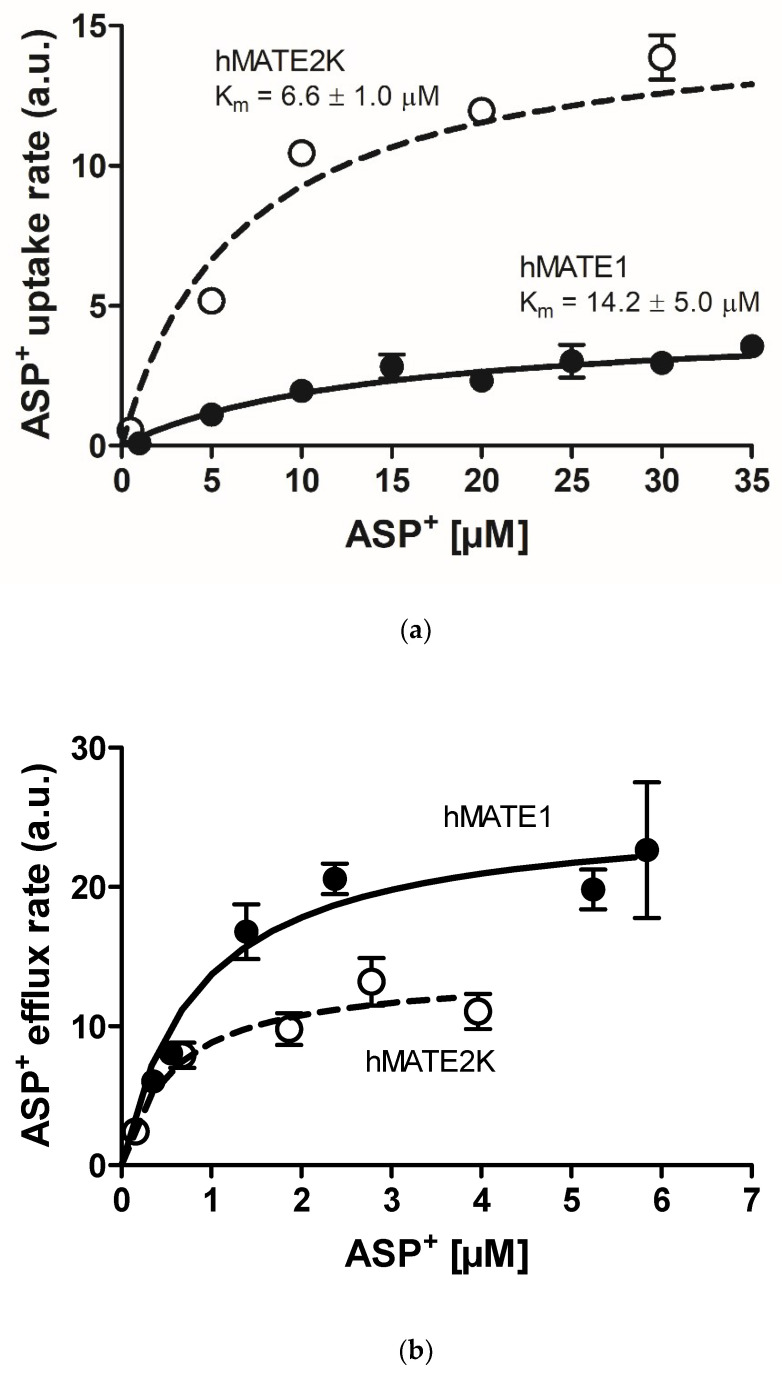
Saturation of 4-(4-dimethylaminostyril)-N-methylpyridinium (ASP^+^) transport mediated by hMATE1 (closed symbols) and hMATE2K (open symbols) in both the uptake (**a**) and efflux conformation (**b**). (**a**) shows the specific ASP^+^ uptake calculated subtracting unspecific (determined in the presence of 1 mM cimetidine) from total ASP^+^ uptake (not shown). (**b**) shows the rate of ASP^+^ efflux calculated as described in Materials and Methods. The calculated K_m_ values are indicated in the figure. The K_m_ values for efflux are not shown, since they can not be exactly calculated because not all the intracellular ASP^+^ is exchangeable. Data are mean ± standard error of the mean (SEM) of three independent experiments, where each ASP^+^ concentration was measured in at least six replicates per experiment. As explained in the text, the V_max_ values are not directly comparable, and for this reason are not shown.

Both human embryonic kidney (HEK) 293 cell lines transfected with either hMATE1 or hMATE2K were able to transport 4-(4-dimethylaminostyril)-N-methylpyridinium (ASP^+^) in a concentration-dependent manner. Specific uptake was calculated by subtraction of unspecific uptake calculated in the presence of 1 mM cimetidine, a high affinity inhibitor of MATEs [20], from total ASP^+^ uptake. Transport reached saturation both in hMATE- and hMATE2K-HEK cells, allowing calculation of the kinetic parameters V_max_ and K_m_, which were, respectively, 4.5 ± 0.7 arbitrary fluorescence units (a.u.) and 14.2 ± 5.0 μM for hMATE1, and 15.3 ± 0.6 a.u. and 6.6 ± 1.0 μM for hMATE2K (Figure 2a, Table 1). The hMATE2K showed a slightly higher affinity for ASP^+^ than hMATE1 in the uptake configuration. The V_max_ values cannot be directly compared, since they also depend on transfection efficiency, which can be different for hMATE1 and hMATE2K, on the gain used in the measurements, and on performance of the fluorimeter lamp. Additionally, in the efflux configuration (Figure 2b), hMATE2K seemed to have a slightly higher affinity for ASP^+^ than hMATE1 (the K_m_ values calculated for efflux are not shown, since they cannot be exactly calculated because not all the intracellular ASP^+^ is exchangeable; however the K_m_ ratio for the efflux mediated by hMATE1/hMATE2K was 1.5, suggesting a higher affinity of hMATE2K than hMATE1 for ASP^+^ in the efflux configuration). Since not all the intracellular ASP^+^ is exchangeable, these values must be considered as an approximation and cannot be directly compared to the K_m_ values measured in the uptake configuration.

**Figure 3:**
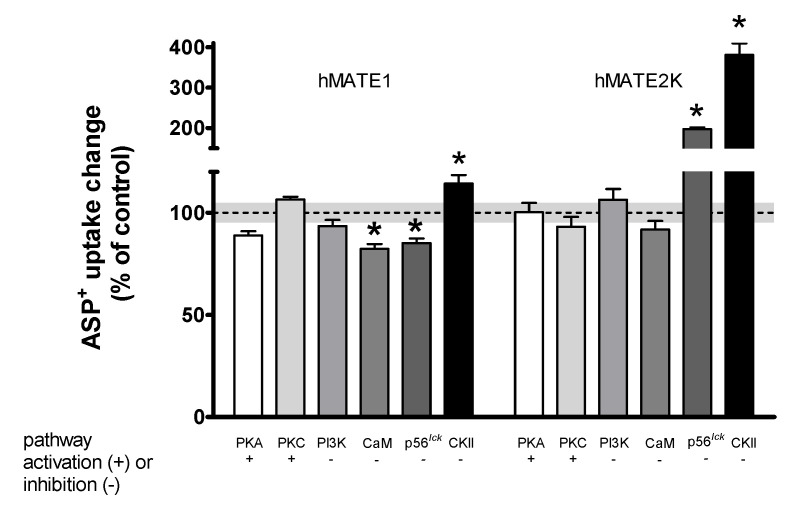
Acute regulation of ASP^+^ uptake in hMATE1- and hMATE2K-HEK293 cells. HEK293 cells expressing hMATE1 or hMATE2K were incubated with a regulator of interest for 10 min before addition of 5 (hMATE1) or 2 (hMATE2K) µM ASP^+^, respectively. Protein kinase A (PKA) was stimulated by incubation with 1 µM forskolin, whereas protein kinase C (PKC) was stimulated with 1 µM 1,2-dioctanoyl-sn-glycerol (DOG). Phosphatidylinositol 3-kinase (PI3K) was inhibited by 0.1 µM wortmannin, Ca^2+^/calmodulin (CaM) by 5 µM calmidazolium, p56^lck^ by 5 µM aminogenistein, and casein kinase II (CKII) by 10 µM 4,5,6,7-tetrabromo-1H-benzimidazole (TBBz). The uptake of ASP^+^ in control wells was set as 100%. All conditions were compared to the control. Each column represents the mean ± SEM with 20–36 replicates measured in at least three independent experiments. The dotted line represents the control value = 100%, with a grey shading representing the SEM variation range. The stars (*) show a statistically significant difference compared to control experiments (*p* < 0.05, unpaired *t*-test).

The rapid regulatory abilities of various cellular signal messengers on ASP^+^ uptake by human MATE transporters are presented in Figure 3. Cells were incubated with each substance for 10 min, before measuring ASP^+^ uptake. For activation of protein kinase A (PKA) and protein kinase C (PKC), we used 1 µM forskolin and 1 µM 1,2-dioctanoyl-sn-glycerol (DOG), respectively. For inhibition of phosphatidylinositol 3-kinase (PI3K), of Ca^2+^/calmodulin (CaM), of p56^lck^ tyrosine kinase (p56^lck^), and of casein kinase II (CKII), we used 0.1 µM wortmannin, 5 µM calmidazolium, 5 µM aminogenistein, and 10 µM 4,5,6,7-tetrabromo-1H-benzimidazole (TBBz), respectively. The regulatory substances were used in a concentration range, which has been described to be specific for activation/inhibition of distinct signaling pathways and which was already used in HEK293 cells to study acute regulation of OCTs [19,21,22,23,24,25,26,27].

The uptake mediated by hMATE1 seemed to be only slightly downregulated by CaM and p56^lck^ inhibition (to −18 ± 2% and −15 ± 2% of controls, respectively). Interestingly, inhibition of CKII stimulated ASP^+^ uptake (+14 ± 4%). Uptake mediated by hMATE2K was strongly stimulated by p56^lck^ and CKII inhibition (to +97 ± 5% and +281 ± 29% of controls, respectively). Other substances (forskolin, DOG, wortmannin) had no significant influence on hMATE-mediated ASP^+^ uptake.

**Figure 4:**
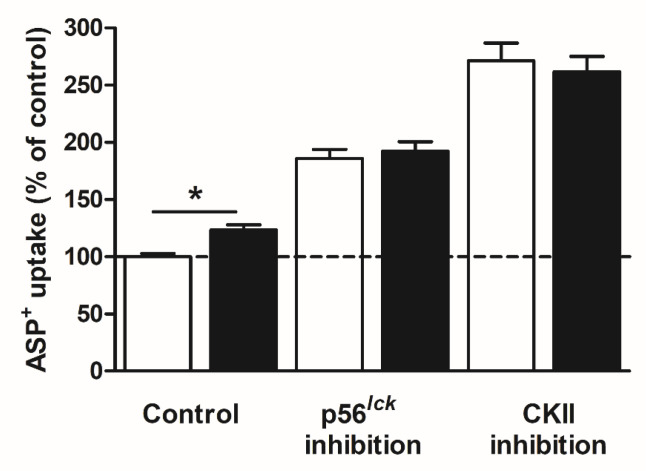
The effect of Na^+^/H^+^ exchanger 1 (NHE1) inhibition with 1 µM cariporide on regulation of ASP^+^ uptake by hMATE2K. hMATE2K-HEK293 cells were incubated with a regulator of interest (p56^lck^ inhibition by 5 µM aminogenistein and CKII inhibition by 10 µM TBBz) alone (open bars) or in the presence of 1 µM cariporide (closed bars) for 10 min. After incubation, 2 μM ASP^+^ solution was applied, and uptake through hMATE2K transporter was measured. Control condition indicates the uptake of ASP^+^ without addition of regulator, which was set to 100%. Results in the presence or absence of cariporide were compared. Each column represents mean ± SEM of at least three independent experiments. The star (*) shows a statistically significant difference compared to experiments without cariporide (*p* < 0.05, unpaired *t*-test).

Because of the huge regulatory effects of p56^lck^ and CKII inhibition on ASP^+^ uptake by hMATE2K, we investigated whether this regulation was due to the activity of Na^+^/H^+^ exchanger 1 (NHE1), which is the main regulator of pH in HEK cells [28]. For this reason, ASP^+^ uptake regulation was measured in hMATE2K-HEK cells under inhibition of NHE1 by 1 µM cariporide, a concentration known to specifically inhibit NHE1 [29] (Figure 4).

**Figure 5:**
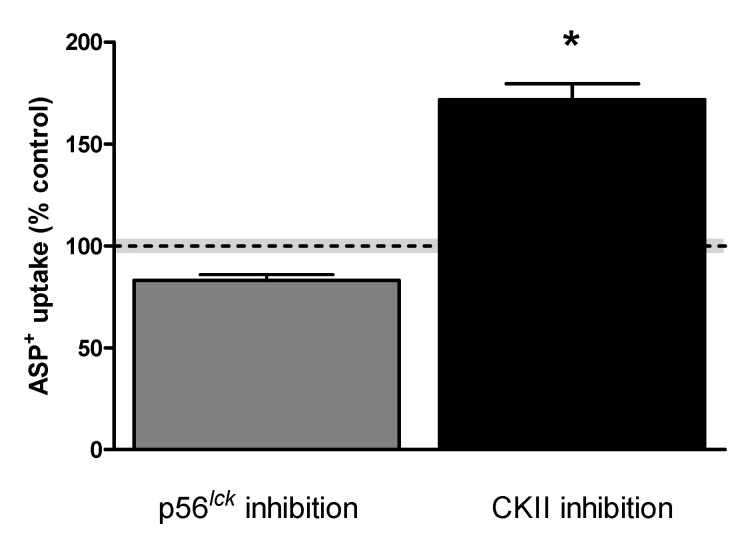
Acute regulation of ASP^+^ uptake into hMATE2K-HEK cells after 30 mM NH_4_Cl pre-treatment to acidify the cells, before 10 min incubation with the regulator of interest (5 µM aminogenistein for p56^lck^ inhibition or 10 µM TBBz for CKII inhibition). After this, incubation solution was replaced with 2 μM ASP^+^, and uptake through hMATE2K was measured. The uptake of ASP^+^ without addition of regulator was set to 100% (dashed line with grey shading representing the SEM variation range). Each column represents mean ± SEM of at least three independent experiments. The star (*) shows a statistically significant difference compared to control experiments without TBBz (*p* < 0.05, unpaired *t*-test).

Ten minutes of incubation with 1 µM cariporide increased ASP^+^ uptake by hMATE2K, probably because of efficient NHE1 inhibition and slight acidification of the cells, which stimulated ASP^+^ uptake. Inhibition of NHE1 with cariporide did not change the stimulation of ASP^+^ uptake by hMATE2K under p56^lck^ or CKII inhibition. To study the effects of pH on regulation of ASP^+^ uptake by hMATE2K, we incubated the cells with NH_4_Cl to make the cellular pH more acidic (Figure S4). This maneuver strongly increased the ASP^+^ uptake by hMATE2K to 292 ± 14% (measured in at least three independent experiments) compared to control experiments, which were set to 100 ± 4% (not shown). Interestingly, under cell acidification, the stimulation of ASP^+^ uptake by hMATE2K observed under p56^lck^ inhibition with aminogenistein disappeared, while the one produced by CKII inhibition with TBBz remained (Figure 5).

### Rapid Regulation of Efflux

**Figure 6:**
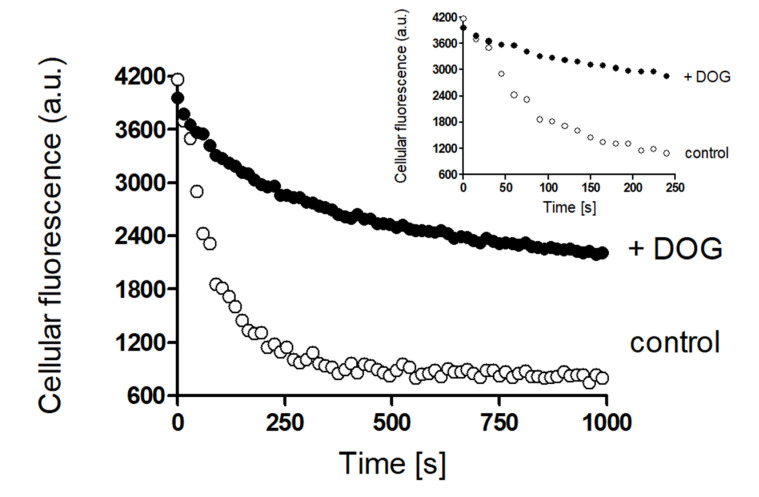
Example of acute regulation of ASP^+^ efflux from hMATE2K-HEK293 cells under PKC activation with 1 µM DOG (closed circles). Prior to addition of DOG, hMATE2K cells were incubated with 25 μM ASP^+^ for 30 min. After that, DOG was added for further 10 min. After this, incubation solution was removed, and cell monolayers were washed two times with ice-cold Ringer-like solution (pH 7.4). Each well was filled with Ringer-like solution and the decrease in fluorescence signal over time was measured. In control experiments, the efflux of ASP^+^ without addition of regulator was measured (open circles). The experimental points of ASP^+^ fluorescence decrease measured in the first 250 s are shown in the insert.

**Figure 7:**
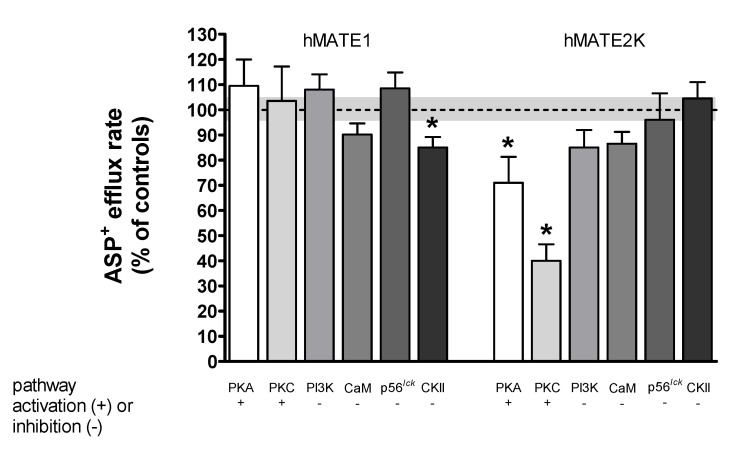
Acute regulation of ASP^+^ efflux from hMATE1- and hMATE2K-HEK293 cells. Prior to the addition of regulator, hMATEs cells were incubated with 25 μM ASP^+^ for 30 min. After that, regulator (PKA was stimulated by incubation with 1 µM forskolin, PKC with 1 µM DOG; PI3K was inhibited by 0.1 µM wortmannin, CaM by 5 µM calmidazolium, p56^lck^ by 5 µM aminogenistein, and CKII by 10 µM TBBz) was added for a further 10 min. After this, incubation solution was removed, and cell monolayers were washed two times with ice-cold Ringer-like solution (pH 7.4). Each well was filled with Ringer-like solution and the decrease in fluorescence signal was measured for 10 min. In control experiments, the efflux of ASP^+^ without addition of regulator was set to 100%. All other conditions were compared with the control. Each column represents mean ± SEM from three different experiments. The dotted line represents the control value = 100%, with the grey shading representing the SEM variation range. The stars (*) show a statistically significant difference compared to control experiments (*p* < 0.05, unpaired *t*-test).

We further investigated whether ASP^+^ efflux mediated by hMATE1 or hMATE2K is also subjected to rapid regulation (Figure 6 and Figure 7). Figure 6 shows an example of hMATE2K efflux regulation experiments performed under PKC activation with DOG compared to control experiments. Figure 7 shows the summary of the results on efflux mediated by hMATE1 and hMATE2K obtained under modulation of several signaling pathways.

**Figure 8:**
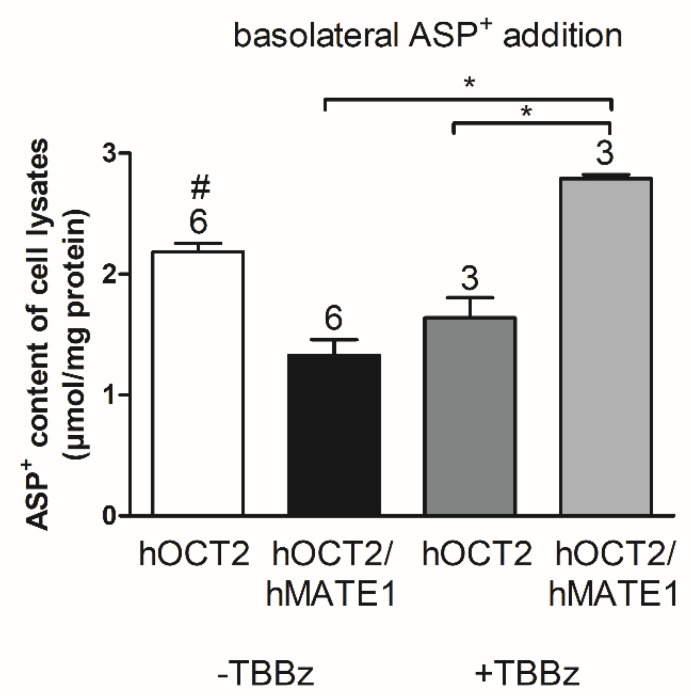
ASP^+^ intracellular accumulation in Madin–Darby canine kidney (MDCK) II cells expressing hOCT2 or hOCT2 together with hMATE1. MDCK II cells expressing the transporters were grown on filters. Upon reaching confluence, we added ASP^+^ (50 µM) to the basolateral compartment, and after 2 h incubation in the presence or absence of 10 µM TBBz, we lysed cells with 4% SDS, with fluorescence in cell lysates being quantified by comparison with cell lysates, where known ASP^+^ concentrations were added. ASP^+^ concentrations in cell lysates were normalized to protein content. Each column represents mean ± SEM from 3–6 different experiments. The stars (*) show a statistically significant difference between the groups and # represents a difference compared to all other groups (*p* < 0.05, ANOVA with Tukey’s post-test).

Since inhibition of CKII with TBBz seems to be able to regulate hMATE1 both in the uptake and in the efflux configuration, we tested whether this regulation can modulate the activity of the transport axis for OCs in Madin–Darby canine kidney (MDCK) II cells, a polarized cell system, where we expressed hOCT2 alone or together with hMATE1. The hMATE1 was transfected in MDCK II cells already stably expressing hOCT2-GFP. The cells were grown on filters (12 well Thin-cert, 1 µm transparent, Greiner Bio-One, Frickenhausen, Germany), allowing the separation of an apical and basolateral compartment. Expression of hOCT2 and hMATE1 was controlled by PCR and immunofluorescence analysis (Figures S7 and S8). The genetic manipulation of MDCK II cells resulted in the expression of hOCT2 and hMATE1 both at the mRNA and at the protein levels. Immunofluorescence labeling of hOCT2 and hMATE1 clearly showed that the two proteins were expressed in distinct cellular compartments, with hOCT2 mainly expressed in the basolateral and hMATE1 in the apical membrane domain. The cellular accumulation of ASP^+^ was compared between hOCT2-MDCK and hOCT2-hMATE1-MDCK cells after addition of the fluorescent substrate to the basolateral compartment in order to mimic the physiological direction of transport in the kidneys (Figure 8). By addition of ASP^+^ to the basolateral compartment, a higher intracellular ASP^+^ accumulation was observed in hOCT2- compared with hOCT2-hMATE1-MDCK-cells, indicating that the presence of hMATE1 increased the ASP^+^ efflux through the apical domain of the plasma membrane. Incubation with TBBz strongly increased the intracellular ASP^+^ content in hOCT2-hMATE1- compared with hOCT2-MDCK-cells, probably due to an inhibition of ASP^+^ efflux through the hMATE1, as observed in experiments with hMATE1-HEK293 cells (Figure 7).

## 3. Discussion

The kidneys deal with rapidly changing quantity of water and solutes, which derives from variable fluid and meal intake and metabolic activities. Transport systems are strongly involved in determining renal function consisting of secretion and reabsorption processes of water and solutes. For this reason, a regulation of transporter activity to cope with different situations is conceivable. Indeed, the renal transport systems are targeted by several hormones, which can initiate a series of regulation pathways [19]. Focusing on renal secretion systems, this regulation has potential pharmacological and pathophysiological importance, since their inhibition may augment the bodily exposure to dangerous synthetic and natural xenobiotics, and their stimulation may be useful for prevention or treatment of pharmacological and occupational renal toxicity [30]. Posttranslational modifications such as phosphorylation/dephosphorylation, glycosylation, and ubiquitination processes are important modulators of protein function, structure, or localization [31]. For transporters, these modifications can alter their kinetic characteristics, such as K_m_ or V_max_ [32].

Considering the renal secretion axis of organic cations (OCs), it is well known that such posttranslational modifications can regulate the first step of secretion, that is, the uptake of OCs into the proximal tubule cells. This process is mainly mediated in humans by hOCT2. From experiments with hOCT2 and also with other orthologs and paralogs, it is well known that glycosylation and oligomerization are important for regulating the insertion of the transporter into the plasma membrane [33,34,35,36] and that multiple intracellular signaling pathways are involved in its rapid regulation [24,27]. Specifically, hOCT2 function was significantly reduced by inhibition of the Ca^2+^/calmodulin complex and stimulation of PKC and of PKA [24]. However, there are only few investigations on the regulation of the final step of renal OC secretion, that is, the transport of OCs by hMATE1 and hMATE2K from proximal tubule cells into the urine. It is conceivable that pathways exist that can regulate renal secretion of organic cations acting both on hOCTs and hMATEs.

The present knowledge on MATE regulation is well summarized in [37]. Transcriptional regulation of MATE1 has been described, together with regulation of its function or mRNA expression under pathological situations such as ischemia/reperfusion injury and diabetes [37]. In inflamed or fibrotic fibroblasts, hMATE expression is downregulated by tumor necrosis factor (TNF)α, interleukin (IL)-16, IL-6, and platelet-derived growth factor (PDGF) [38,39], and stimulated by the Notch pathway [39]. In plasma membranes from human kidney cortex, the protein expression of hMATE was found to not be gender- or age-dependent, at least in renal samples from adults [40].

To increase the knowledge on hMATE regulation and find out whether there is a regulation of the OC secretory axis, we investigated in this work the hMATE rapid regulation, focusing on pathways that are known to modulate hOCT function.

Physiologically, hMATEs work as OC/H^+^ exchangers, driven by the slight acidity of primary urine in proximal tubules. Working in vitro with HEK293 cells as an expression system for hMATEs, we can study the transport characteristics of the transporters, offering to the cells substrates from the extracellular site and investigating their uptake by hMATEs, which is driven by the negative membrane potential. This uptake can be stimulated by decreasing the intracellular pH. The HEK system can be also used to study the function of hMATEs as OC efflux transporters by loading the cells with substrates and measuring their efflux kinetics.

Using the fluorescent organic cation ASP^+^ as a substrate, we showed that both ASP^+^ uptake and efflux were pH-dependent (Figure S5) and that hMATE2K has a slightly higher affinity than hMATE1 for the substrate both in the efflux and in the uptake configuration. Performing efflux experiments over 30 min, we measured that up to 40% (41 ± 6%, *N* = 4, not shown) of ASP^+^ is not exchangeable. For this reason, the K_m_ for ASP^+^ in the efflux configuration cannot be exactly determined.

**Figure 9:**
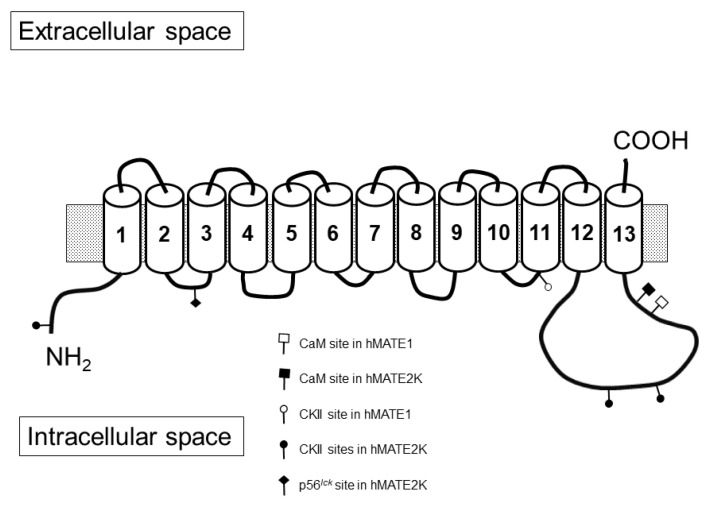
This figure shows a schematic secondary structure of hMATEs, as determined using the eukaryotic linear motif resource [43]. Both hMATE1 (NP_060712.2, NM_018242.3) and hMATE2K (NP_001093116.1, NM_001099646.2) are modelled as having 13 transmembrane domains (TMD), with intracellular amino- and extracellular carboxy-termini. Potential CaM, CKII, and p56^lck^ phosphorylation sites identified as explained in the Materials and Methods section are also shown. Several other potential phosphorylation sites for PKA (S23/S119/S249/S335/S336/T337/S538 in hMATE1; S469/S508/S534 in hMATE2K) and for PKC (T98/S101/T103/S335/S336/S538 in hMATE1; T325/T398/S469/S492/T496/S498/T505/T506/T530/S531/S534 in hMATE2K) were identified using GPS 5.0 [41], but for the sake of clarity, they are not indicated in the figure.

Rapid regulation of ASP^+^ uptake was studied first. Here, hMATE1 activity was decreased by inhibition of the Ca^2+^/calmodulin complex with calmidazolium and of p56^lck^ tyrosine kinase with aminogenistein, while inhibition of CKII with TBBz stimulated hMATE1-mediated ASP^+^ uptake, indicating that in HEK293 cells these pathways are endogenously active and regulate hMATE1 uptake. A search of putative calmodulin binding sites in hMATE1 using the calmodulin binding database of the Ikura Lab, Ontario Cancer Institute (http://calcium.uhnres.utoronto.ca/ctdb/ctdb/home.html), showed that it has a putative calmodulin binding sequence at position 533 (DGAKLSRK). Using the group-based prediction system GPS 5.0 [41], in the putative intracellular domains of hMATE1 amino acid sequence (Table S2), no direct p56^lck^ phosphorylation site could be identified, while a CKII potential phosphorylation site was detected at position S402 (Figure 9). ASP^+^ uptake by hMATE2K was significantly stimulated by inhibition of p56^lck^ tyrosine kinase and of CKII. While CKII-induced regulation was stronger than that of the same type as was measured for hMATE1, the effect of p56^lck^ tyrosine kinase was opposite to what was observed for hMATE1. This strong stimulation of hMATE2K by inhibition of p56^lck^ tyrosine kinase and of CKII was not dependent on a changed activity of NHE1, since it was still present when the experiments were performed under NHE1 inhibition. However, the regulation by inhibition of p56^lck^ tyrosine kinase disappeared under cellular acidification, suggesting that it involves some interaction close to the H^+^ binding site, probably in proximity of the N-terminus, where H^+^-driven conformational changes of MATEs take place [42]. Indeed, a potential phosphorylation site for p56^lck^ was present in the putative second intracellular loop of hMATE2K at position Y104 (Figure 9). CKII has putative phosphorylation sites at S3, S508, and S519 (Figure 9).

For what concerns regulation of ASP^+^ efflux, inhibition of CKII caused a downregulation of efflux by hMATE1, in clear opposition to what observed for uptake, suggesting that incubation with TBBz stabilizes hMATE1 in an uptake configuration. In hMATE2K cells, this maneuver did not change the efflux of ASP^+^, in strong contrast to what measured for ASP^+^ uptake, also here suggesting that TBBz stabilizes the transporter rather than an uptake configuration. The other pathways did not change ASP^+^ efflux, except for that concerning activation of PKA with forskolin and of PKC with DOG, which in hMATE2K cells significantly inhibited its function, suggesting that PKA and PKC inhibit the efflux by hMATE2K. A search of putative phosphorylation sites in hMATE2K showed that it has several potential PKA and PKC phosphorylation sites in intracellular domains (Table S2), suggesting that these sites may be the target of this regulation.

Can a regulation of the transporter axis change the renal secretion of substrates? This point was investigated in MDCK II cells, a polarized cell model, resembling the physiological polarization of renal tubules, where the hOCT2 alone or together with hMATE1 were expressed. Expression of hMATE1 reduced the cellular ASP^+^ accumulation, probably by increasing its transport out of cells. Interestingly, inhibition of CKII resulted in a higher cellular ASP^+^ content, confirming the results obtained with HEK293 cells, where incubation with TBBz reduced its efflux. For this reason, regulation of transporters involved in renal secretion of organic cations may be an approach to stimulate renal secretion or to decrease exposition of kidney cells to nephrotoxicants.

Comparing these results with the data from the literature, we found hOCT2 to be regulated by several different signaling pathways [24,27,44], while the activity of hMATEs both in the uptake and in the efflux configuration was found to be regulated only by few pathways (Table 2).

## 4. Materials and Methods

Cell culture: Human embryonic kidney (HEK) 293 cells stably expressing hMATE1 or hMATE2K or the respective empty vector were used for the experiments. Generation of these cell lines has been already described elsewhere [45]. HEK293 cells were maintained at 37 °C, 5% CO_2_, in 50 mL cell culture flasks (Greiner, Frickenhausen, Germany). Cell medium consisted of Dulbecco’s minimal Eagle’s medium (Biochrom, Berlin, Germany) supplemented with 10% fetal bovine serum, 1 g/L glucose, 2 mM glutamine, 3.7 g/L NaHCO_3_, and 100 U/mL streptomycin/penicillin (Biochrom). Selection of cells transfected with hMATE1 or hMATE2K transporter was assured by the addition of hygromycin (200 or 175 mg/mL, respectively). Cell cultures were grown on 96-, 24-, or 12-well plates until 80–90% confluence was reached. Experiments were performed with cells from passages 40–65. A brief characterization of these cell lines is shown in the Figures S1–S3.

For some experiments, the MDCK II cell line was used, since it has a clear apical-basolateral polarity, well-defined cell junctions, and a rapid growth rate, and because it polarizes in cell culture [46]. MDCK II cells were transduced with hOCT2-GFP inserted into the vector pQCXIH (Clontech Laboratories, Takara Bio USA, Mountain View, CA, USA) by a retroviral transduction technique, as described in [47]. These cells were then transiently transfected with hMATE1 inserted into the pcDNA 3.1 vector [16] (a kind gift by Atushi Yonezawa, Kyoto University, Japan). Transfection was performed with Lipofectamine 2000 transfection reagent according to the manufacturer’s instructions (Fisher Scientific, Schwerte, Germany).

Reagents: ASP^+^ was purchased from Fischer Scientific. Wortmannin, calphostin C, calmidazolium, and aminogenistein were purchased from Calbiochem (Calbiochem, Merck Chemicals, Darmstadt, Germany). All other reagents were of the highest purity and were obtained from Sigma-Aldrich (Sigma-Aldrich, Merck Chemicals, Darmstadt, Germany).

Fluorescence measurements.: The fluorescent organic cation ASP^+^ was used to monitor hMATE activity, as already performed in other works [45,48]. Measurements were performed using a microplate fluorescence reader with excitation at 465 nm and emission at 590 nm (Infinite F200, Tecan, Switzerland), as already described in detail [23].

Transport characteristics and acute regulation of hMATE1 and hMATE2K were studied using two different protocols. The first one aimed to test function and acute regulation of hMATEs in the uptake configuration, and the second one aimed to measure these parameters in an efflux configuration.

**Figure 10:**
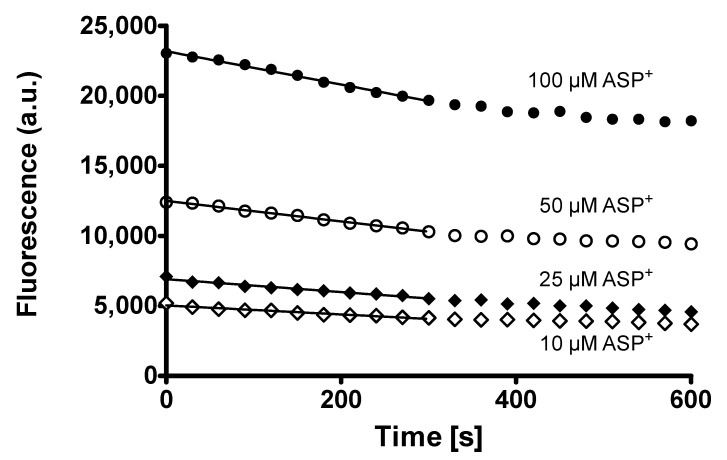
Example of efflux experiments performed after 10 min incubation of hMATE1-HEK cells with 10–100 µM ASP^+^ at 37 °C. The decrease of cellular fluorescence upon time in seconds is shown for different ASP^+^ concentrations used. The lines show the part of the fluorescence decrease used to calculate the slope of efflux velocity. Fluorescence is given as arbitrary units (a.u.).

Before measurements, cell monolayers were washed with Ringer-like solution containing (in mM): NaCl 145, K_2_HPO_4_ 1.6, KH_2_PO_4_ 0.4, D-glucose 5, MgCl_2_ 1, and calcium gluconate 1.3, with pH adjusted to 7.4 at 37 °C. For uptake kinetic experiments, OC transport was measured dynamically at 37 °C after addition of ASP^+^ in a 1–35 µM concentration range as initial rate of fluorescence increase [23]. Slopes of fluorescence increase were linearly fitted and used as ASP^+^ uptake measure. For measurements of transporter activity in the efflux configuration, we incubated cells for 10 min at 37 °C with ASP^+^ in a 10–100 µM concentration range. After this incubation, cells were washed with ice-cold Ringer-like solution and the decrease of fluorescence was measured at 37 °C for 10 min. The slope of the initial fluorescence decrease was used as a measure of transporter efflux velocity. This part of the efflux seemed to be mediated mainly by hMATEs (Figure S3). To calibrate for cellular ASP^+^ content at the beginning of efflux measurements, in unpaired experiments after incubation with ASP^+^ and washing with ice-cold Ringer-like solution, we lysed cells with 4% sodium dodecyl sulfate (SDS) in 10 mM Tris-HCl (pH 7.4) and their fluorescence was compared with that measured in SDS cell lysates, where known ASP^+^ concentrations were added, as explained in Figure S9. An example of the fluorescence decrease in efflux experiments is given in Figure 10. In both uptake and efflux experiments, we also evaluated pH dependence of ASP^+^ transport by hMATEs (Figure S5).

In some experiments, the dependence of ASP^+^ uptake by hMATEs on pH was investigated using intracellular acidification. To do this, cells were incubated with 30 mM NH_4_Cl for 30 min and, before addition of ASP^+^, the NH_4_Cl solution was replaced by fresh Ringer-like solution (37 °C, pH 7.4). The course of pH changes induced by this procedure was investigated in unpaired experiments using 2′,7′-bis(2-carboxyethyl)-5(6)-carboxyfluorescein acetoxymethyl ester (BCECF-AM). BCECF-AM is a membrane permeable substance, which functions as pH-sensitive fluorescent dye [49,50]. Briefly, confluent hMATE-HEK cells were incubated in the dark with Ringer-like solution containing 5 μM BCECF-AM for 30 min. After incubation, the dye was removed, and the cells were incubated with Ringer-like solution with or without 30 mM NH_4_Cl for 30 min. After this time, incubation solution was replaced by fresh Ringer-like buffer. All the solutions had a pH 7.4 at 37 °C. The pH changes were monitored with Tecan Infinite M200 (Tecan, Switzerland) by ratiometric measurements of BCECF fluorescence emission at 540 nm after excitation at the wavelengths of 440 nm (isosbestic point) and 490 nm (pH-dependent) (Figure S4).

Acute regulation of ASP^+^ transport by hMATEs was studied in the uptake and in the efflux transporter configuration. Regulation of ASP^+^ uptake was studied after 10 min incubation with known regulators of important signaling pathways, which are known to be active in HEK293 cells [19,21,23,24,26,27]. The concentrations of the potential regulators were chosen according to previous regulation studies of OCT function [19,21,23,24,26,27]. After pre-treatment with the potential regulator, ASP^+^ was added to the cells and its uptake over time was monitored. In some experiments, cells were treated with NH_4_Cl to induce an acidification and were then incubated 10 min with the regulator before replacing incubation solution with Ringer-like buffer and measuring ASP^+^ uptake.

For regulation experiments of hMATE-mediated ASP^+^ efflux, we loaded confluent hMATEs-HEK293 cells with 25 µM ASP^+^ for 30 min. After that, regulator of interest or Ringer-like solution as a control was applied for 10 more minutes, still in the presence of ASP^+^. Afterwards, incubation solution was removed, each well was washed with ice-cold Ringer-like solution, and the decrease in fluorescence was measured at 37 °C, as described above.

The effect of each regulator on ASP^+^ uptake and efflux were compared with control conditions without potential regulator, which were set as 100%. In order to test the specificity of regulatory effects, we measured regulation by some effective substance also in the presence of 10 µM cariporide, an inhibitor of NHE1 [29].

Immunofluorescence analysis: MDCK-hOCT2-GFP cells transfected with hMATE1 and growing on filters for 7 days were fixed in 4% paraformaldehyde (PFA). After fixation, the cells were washed three times with Dulbecco’s phosphate-buffered saline (PBS, Biochrom, Berlin, Germany) and permeabilized using 0.1% Triton X-100 for 5 min. After extensive washing with PBS, unspecific binding sites were blocked by overnight incubation at 4 °C with 10% bovine serum albumin (BSA, Sigma-Aldrich) in PBS. Cells were then incubated 60 min at room temperature with a rabbit anti-hMATE1 antibody (hMATE1 E13, sc-133390, Santa Cruz, Dallas, TX, USA) diluted 1:10 in 1% BSA in PBS. After three washing steps in PBS, the secondary antibody (anti-rabbit IgG Alexa Fluor 594, Cell Signaling, Frankfurt/Main, Germany) at a 1:1.000 dilution was incubated for 60 min followed by five more washing steps in PBS. The nuclei were blue-labeled with 2-(4-amidinophenyl)-1H-indole-6-carboxamidine (DAPI, Sigma-Aldrich). Finally, cells were covered with Fluoromount (Sigma-Aldrich), and fluorescence photographs were taken by epifluorescence microscopy (Observer Z1 with apotome, Zeiss, Göttingen, Germany). Negative control slides were included without addition of primary antibody (data not shown).

PCR analysis: For PCR analysis of MDCK II cells, total RNA from wild type (WT) cells or cells expressing hOCT2 alone or together with hMATE1 or only the empty vectors (EV) were isolated using the Qiagen RNeasy Midikit (Qiagen, Gilden, Germany) and reverse transcription was performed using the Superscript II system (Invitrogen, Carlsbad, CA), both according to the manufacturer’s recommendations. Standard PCR was performed using specific primer pairs as listed in Table S1. The PCR products were separated using agarose gel electrophoresis.

Statistical analysis: Experimental data are presented as means ± SEM, with *n* referring to the number of totally measured replicates obtained in at least three independent experiments. Significant differences of regulatory substances were calculated using unpaired Student’s *t*-test or ANOVA with Tukey’s post-test for multiple comparisons. A *p*-value < 0.05 was considered statistically significant. Analyses were performed using GraphPad Prism, Version 5.3 (GraphPad Software, San Diego, CA, USA).

## 5. Conclusions

As found for other SLC transporter families, hMATEs, members of the renal secretion pathway of organic cations, can be acutely regulated. Although hMATEs are highly similar in their structure and substrate affinities, they do have some differences for what concerns responses to cellular messengers. Preliminary results suggest that such a regulation is effective in systems expressing uptake and extrusion transporters.

## Figures and Tables

**Table 1:** This table summarizes the affinities (K_m_ in µM) of hMATE1 and hMATE2K for ASP^+^ measured in the uptake configuration.

Transport Direction	Transporter Affinity (K_m_)	
	hMATE1	hMATE2K
Uptake	14.2 ± 5.0 µM	6.6 ± 1.0 µM

**Table 2:** Signaling pathways and their effects on hOCT2-, hMATE1-, and hMATE2K-mediated transport.

Signaling Pathway	hOCT2 Uptake	hMATE1 Uptake	hMATE1 Efflux	hMATE2K Uptake	hMATE2K Efflux
PKA	↓[24]	0	0	0	↓
PKC	↓[24]	0	0	0	↓
CKII	↑	↓	↑	↓	0
CaM	↑[24]	↑	0	0	0
p56^lck^	↑[44]	↑	0	↓	0
PI3K	↓[24]	0	0	0	0

0 = no effect; ↑/↓ = the activity of the indicated kinase stimulates/inhibits the transporters. When no reference is indicated, the results refer to the present work.

## Supplementary Materials

Supplementary materials can be found at https://www.mdpi.com/1422-0067/21/14/5157/s1.

## Abbreviations

ASP^+^	4-(4-(dimethylamino)styryl)-N-methylpyridinium
a.u.	arbitrary units
BCECF-AM	2′,7′-bis(2-carboxyethyl)-5(6)-carboxyfluorescein acetoxymethyl ester
BSA	bovine serum albumin
CaM	calmodulin
CKII	casein-kinase II
DAPI	2-(4-amidinophenyl)-1H-indole-6-carboxamidine
DOG	1,2-dioctanoyl-sn-glycerol
HEK	human embryonic kidney
hMATE	human multidrug and toxins extrusion protein
hOCT	human organic cation transporter
IL	interleukin
MDCK	Madin–Darby canine kidney
NHE1	Na^+^/H^+^ exchanger 1
OCs	organic cations
PBS	phosphate-buffered saline
PDGF	platelet-derived growth factor
PFA	paraformaldehyde
PI3K	phosphatidylinositol 3-kinase
PKA	protein kinase A
PKC	protein kinase C
p56^lck^	p56^lck^ tyrosine kinase
SDS	sodium dodecyl sulfate
SEM	standard error of the mean
TBBz	4,5,6,7-tetrabromo-1H-benzimidazole
TNF	tumor necrosis factor

## References

[1] Koepsell H. (2004). Polyspecific organic cation transporters: Their functions and interactions with drugs. Trends Pharmacol. Sci..

[2] Wright S.H. (2005). Role of organic cation transporters in the renal handling of therapeutic agents and xenobiotics. Toxicol. Appl. Pharmacol..

[3] Wright S.H., Dantzler W.H. (2004). Molecular and cellular physiology of renal organic cation and anion transport. Physiol. Rev..

[4] Wagner D.J., Hu T., Wang J. (2016). Polyspecific organic cation transporters and their impact on drug intracellular levels and pharmacodynamics. Pharmacol. Res..

[5] Lai R.E., Jay C.E., Sweet D.H. (2018). Organic solute carrier 22 (SLC22) family: Potential for interactions with food, herbal/dietary supplements, endogenous compounds, and drugs. J. Food Drug Anal..

[6] Motohashi H., Sakurai Y., Saito H., Masuda S., Urakami Y., Goto M., Fukatsu A., Ogawa O., Inui K.K. (2002). Gene expression levels and immunolocalization of organic ion transporters in the human kidney. J. Am. Soc. Nephrol..

[7] Koepsell H., Lips K., Volk C. (2007). Polyspecific organic cation transporters: Structure, function, physiological roles, and biopharmaceutical implications. Pharm. Res..

[8] Otsuka M., Matsumoto T., Morimoto R., Arioka S., Omote H., Moriyama Y. (2005). A human transporter protein that mediates the final excretion step for toxic organic cations. Proc. Natl. Acad. Sci. USA.

[9] Terada T., Inui K. (2008). Physiological and pharmacokinetic roles of H^+^/organic cation antiporters (MATE/SLC47A). Biochem. Pharmacol..

[10] Masuda S., Terada T., Yonezawa A., Tanihara Y., Kishimoto K., Katsura T., Ogawa O., Inui K. (2006). Identification and functional characterization of a new human kidney-specific H^+^/organic cation antiporter, kidney-specific multidrug and toxin extrusion 2. J. Am. Soc. Nephrol..

[11] Komatsu T., Hiasa M., Miyaji T., Kanamoto T., Matsumoto T., Otsuka M., Moriyama Y., Omote H. (2011). Characterization of the human MATE2 proton-coupled polyspecific organic cation exporter. Int. J. Biochem. Cell Biol..

[12] Yonezawa A., Inui K. (2011). Importance of the multidrug and toxin extrusion MATE/SLC47A family to pharmacokinetics, pharmacodynamics/toxicodynamics and pharmacogenomics. Br. J. Pharmacol..

[13] Zhang X., Wright S.H. (2009). MATE1 has an external COOH terminus, consistent with a 13-helix topology. Am. J. Physiol Renal Physiol..

[14] König J., Zolk O., Singer K., Hoffmann C., Fromm M.F. (2011). Double-transfected MDCK cells expressing human OCT1/MATE1 or OCT2/MATE1: Determinants of uptake and transcellular translocation of organic cations. Br. J. Pharmacol..

[15] Inui K.I., Masuda S., Saito H. (2000). Cellular and molecular aspects of drug transport in the kidney. Kidney Int..

[16] Sato T., Masuda S., Yonezawa A., Tanihara Y., Katsura T., Inui K. (2008). Transcellular transport of organic cations in double-transfected MDCK cells expressing human organic cation transporters hOCT1/hMATE1 and hOCT2/hMATE1. Biochem. Pharmacol..

[17] Yonezawa A., Inui K. (2011). Organic cation transporter OCT/SLC22A and H^+^/organic cation antiporter MATE/SLC47A are key molecules for nephrotoxicity of platinum agents. Biochem. Pharmacol..

[18] Yokoo S., Yonezawa A., Masuda S., Fukatsu A., Katsura T., Inui K. (2007). Differential contribution of organic cation transporters, OCT2 and MATE1, in platinum agent-induced nephrotoxicity. Biochem. Pharmacol..

[19] Ciarimboli G., Schlatter E. (2005). Regulation of organic cation transport. Pflügers Arch..

[20] Matsushima S., Maeda K., Inoue K., Ohta K.Y., Yuasa H., Kondo T., Nakayama H., Horita S., Kusuhara H., Sugiyama Y. (2009). The inhibition of human multidrug and toxin extrusion 1 is involved in the drug-drug interaction caused by cimetidine. Drug Metab. Dispos..

[21] Massmann V., Edemir B., Schlatter E., Al-Monajjed R., Harrach S., Klassen P., Holle S.K., Sindic A., Dobrivojevic M., Pavenstadt H. (2014). The organic cation transporter 3 (OCT3) as molecular target of psychotropic drugs: Transport characteristics and acute regulation of cloned murine OCT3. Pflügers Arch..

[22] Schlatter E., Klassen P., Massmann V., Holle S.K., Guckel D., Edemir B., Pavenstädt H., Ciarimboli G. (2014). Mouse organic cation transporter 1 determines properties and regulation of basolateral organic cation transport in renal proximal tubules. Pflügers Arch..

[23] Wilde S., Schlatter E., Koepsell H., Edemir B., Reuter S., Pavenstädt H., Neugebauer U., Schröter R., Brast S., Ciarimboli G. (2009). Calmodulin-associated post-translational regulation of rat organic cation transporter 2 in the kidney is gender dependent. Cell. Mol. Life Sci..

[24] Biermann J., Lang D., Gorboulev V., Koepsell H., Sindic A., Schröter R., Zvirbliene A., Pavenstädt H., Schlatter E., Ciarimboli G. (2006). Characterization of regulatory mechanisms and states of human organic cation transporter 2. Am. J. Physiol. Cell Physiol..

[25] Ciarimboli G., Koepsell H., Iordanova M., Gorboulev V., Dürner B., Lang D., Edemir B., Schröter R., van Le T., Schlatter E. (2005). Individual PKC-phosphorylation sites in organic cation transporter 1 determine substrate selectivity and transport regulation. J. Am. Soc. Nephrol..

[26] Ciarimboli G., Struwe K., Arndt P., Gorboulev V., Koepsell H., Schlatter E., Hirsch J.R. (2004). Regulation of the human organic cation transporter hOCT1. J. Cell Physiol..

[27] Cetinkaya I., Ciarimboli G., Yalcinkaya G., Mehrens T., Velic A., Hirsch J.R., Gorboulev V., Koepsell H., Schlatter E. (2003). Regulation of human organic cation transporter hOCT2 by PKA, PI3K, and calmodulin-dependent kinases. Am. J. Physiol. Renal Physiol..

[28] Willoughby D., Masada N., Crossthwaite A.J., Ciruela A., Cooper D.M. (2005). Localized Na^+^/H^+^ exchanger 1 expression protects Ca^2+^-regulated adenylyl cyclases from changes in intracellular pH. J. Biol. Chem..

[29] Dhein S., Salameh A. (1999). Na^+^/H^+^-exchange inhibition by cariporide (Hoe 642): A new principle in cardiovascular medicin. Cardiovasc. Drug Rev..

[30] Berkhin E.B., Humphreys M.H. (2001). Regulation of renal tubular secretion of organic compounds. Kidney Int.

[31] Czuba L.C., Hillgren K.M., Swaan P.W. (2018). Post-translational modifications of transporters. Pharmacol. Ther..

[32] Xu D., You G. (2017). Loops and layers of post-translational modifications of drug transporters. Adv. Drug Deliv. Rev..

[33] Keller T., Egenberger B., Gorboulev V., Bernhard F., Uzelac Z., Gorbunov D., Wirth C., Koppatz S., Dotsch V., Hunte C. (2011). The large extracellular loop of organic cation transporter 1 influences substrate affinity and is pivotal for oligomerization. J. Biol. Chem..

[34] Pelis R.M., Suhre W.M., Wright S.H. (2006). Functional influence of N-glycosylation in OCT2-mediated tetraethylammonium transport. Am. J. Physiol. Renal. Physiol..

[35] Pelis R.M., Zhang X., Dangprapai Y., Wright S.H. (2006). Cysteine accessibility in the hydrophilic cleft of the human organic cation transporter 2. J. Biol. Chem..

[36] Brast S., Grabner A., Sucic S., Sitte H.H., Hermann E., Pavenstädt H., Schlatter E., Ciarimboli G. (2012). The cysteines of the extracellular loop are crucial for trafficking of human organic cation transporter 2 to the plasma membrane and are involved in oligomerization. FASEB J..

[37] Nies A.T., Damme K., Kruck S., Schaeffeler E., Schwab M. (2016). Structure and function of multidrug and toxin extrusion proteins (MATEs) and their relevance to drug therapy and personalized medicine. Arch. Toxicol..

[38] Schmidt-Lauber C., Harrach S., Pap T., Fischer M., Victor M., Heitzmann M., Hansen U., Fobker M., Brand S.M., Sindic A. (2012). Transport mechanisms and their pathology-induced regulation govern tyrosine kinase inhibitor delivery in rheumatoid arthritis. PLoS ONE.

[39] Harrach S., Barz V., Pap T., Pavenstädt H., Schlatter E., Edemir B., Distler J., Ciarimboli G., Bertrand J. (2019). Notch signaling activity determines uptake and biological effect of Imatinib in systemic sclerosis dermal fibroblasts. J. Investig. Dermatol..

[40] Oswald S., Muller J., Neugebauer U., Schroter R., Herrmann E., Pavenstadt H., Ciarimboli G. (2019). Protein abundance of clinically relevant drug transporters in the human kidneys. Int. J. Mol. Sci..

[41] Xue Y., Ren J., Gao X., Jin C., Wen L., Yao X. (2008). GPS 2.0, a tool to predict kinase-specific phosphorylation sites in hierarchy. Mol. Cell Proteomics.

[42] Claxton D.P., Jagessar K.L., Steed P.R., Stein R.A., Mchaourab H.S. (2018). Sodium and proton coupling in the conformational cycle of a MATE antiporter from *Vibrio cholerae*. Proc. Natl. Acad. Sci. USA.

[43] Gouw M., Michael S., Samano-Sanchez H., Kumar M., Zeke A., Lang B., Bely B., Chemes L.B., Davey N.E., Deng Z. (2018). The eukaryotic linear motif resource—2018 update. Nucleic. Acids Res..

[44] Frenzel D., Köppen C., Bauer O.B., Karst U., Schröter R., Tzvetkov M.V., Ciarimboli G. (2019). Effects of single nucleotide polymorphism Ala270Ser (rs316019) on the function and regulation of hOCT2. Biomolecules.

[45] Hucke A., Park G.Y., Bauer O.B., Beyer G., Köppen C., Zeeh D., Wehe C.A., Sperling M., Schröter R., Kantauskaite M. (2018). Interaction of the new monofunctional anticancer agent Phenanthriplatin with transporters for organic cations. Front. Chem..

[46] Dukes J.D., Whitley P., Chalmers A.D. (2011). The MDCK variety pack: Choosing the right strain. BMC Cell Biol..

[47] Schulze U., Vollenbröker B., Braun D.A., Le T.V., Granado D., Kremerskothen J., Fränzel B., Klosowski R., Barth J., Fufezan C. (2014). The Vac14-interaction network is linked to regulators of the endolysosomal and autophagic pathway. Mol. Cell Proteomics.

[48] Wittwer M.B., Zur A.A., Khuri N., Kido Y., Kosaka A., Zhang X., Morrissey K.M., Sali A., Huang Y., Giacomini K.M. (2013). Discovery of potent, selective multidrug and toxin extrusion transporter 1 (MATE1, SLC47A1) inhibitors through prescription drug profiling and computational modeling. J. Med. Chem..

[49] Bright G.R., Fisher G.W., Rogowska J., Taylor D.L. (1987). Fluorescence ratio imaging microscopy: Temporal and spatial measurements of cytoplasmic pH. J. Cell Biol..

[50] Grant R.L., Acosta D. (1997). Ratiometric measurement of intracellular pH of cultured cells with BCECF in a fluorescence multi-well plate reader. In Vitro Cell Dev. Biol. Anim..

