# Supplementary material for: Rapid Regulation of Human Multidrug and Extrusion Transporters hMATE1 and hMATE2K
Source: Int J Mol Sci. 2020 Jul 21;21(14):5157. doi: 10.3390/ijms21145157 (PMC7404265; doi:10.3390/ijms21145157)
Supplement: Supplementary file 1 [file ijms-21-05157-s001.pdf]

Rapid regulation of human multidrug and extrusion transporters hMATE1 and hMATE2K

Marta Kantauskaite[1*], Anna Hucke[1*], Moritz Reike[1], Sara Ahmed Eltayeb[1], Chuyan Xiao[1], Vivien Barz[1], Giuliano Ciarimboli[1#]

Supplementary Materials

Supplementary Materials Table 1

Sense (S) and antisense (AS) primers for RT-PCR (5‘ to 3‘) of human (h) and dog (d) OCT2 and GAPDH, and hMATE1 in MDCK II cells

hOCT2	S	TCATGGCCATTTCCCAACC
	AS	CACCAGGAGCCCAACTGTAT
hMATE1	S	AAGCTGGAGCTGGATGCAGTC
	AS	CAGCAGAGGAGCAGGACGAGC
hGAPDH	S	CAAGCTCATTTCCTGGTATGAC
	AS	GTGTGGTGGGGGACTGAGTGTGG
dOCT2	S	GGCTACCTAGCAGACAGGTTT
	AS	GATAGCTCCGCCCGACAAAT
dGAPDH	S	CCCACTCTTCCACCTTCGAC
	AS	TTCTCAGCCTTGACTGTGCC

Supplementary Materials Table 2. Transmembrane domains (TMD) regions of hMATE1 and hMATE2K as determined using Protter - visualize proteoforms (Omasits, U.; Ahrens,C.H.; Muller,S.; Wollscheid,B. Protter: interactive protein feature visualization and integration with experimental proteomic data. Bioinformatics 2014, 30, 884-886).

	hMATE1 (NM_018242.3) 570 aa		hMATE2K (NM_001099646.2) 566 aa	
TMD	Start	End	Start	End
1	37	59	31	53
2	74	96	68	90
3	121	143	116	138
4	153	175	153	171
5	182	204	184	206
6	214	236	211	233
7	257	276	254	273
8	296	318	288	310
9	338	356	331	353
10	371	388	373	395
11	408	430	404	426
12	435	457	431	453
13	548	567	542	564

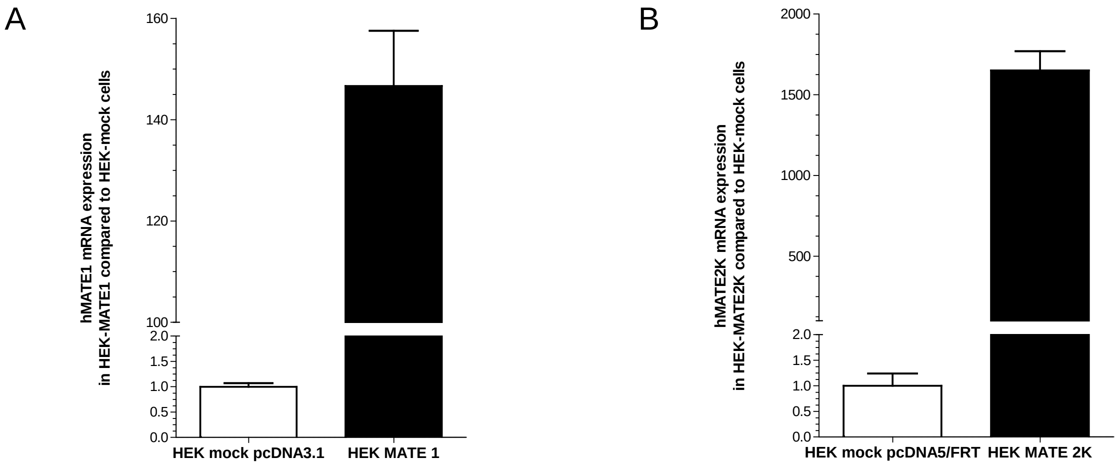

Supplementary Materials Figure 1. mRNA expression for the respective transporter in hMATE1- (panel A), hMATE2K- (panel B), and mock-transfected cells measured by quantitative PCR: The mRNA-expression of hMATE1 and hMATE2K in cells transfected with the transporters was 150- and 1600-times higher than in mock-cells, respectively. The mean values ± SEM from 3 independent experiments are shown.

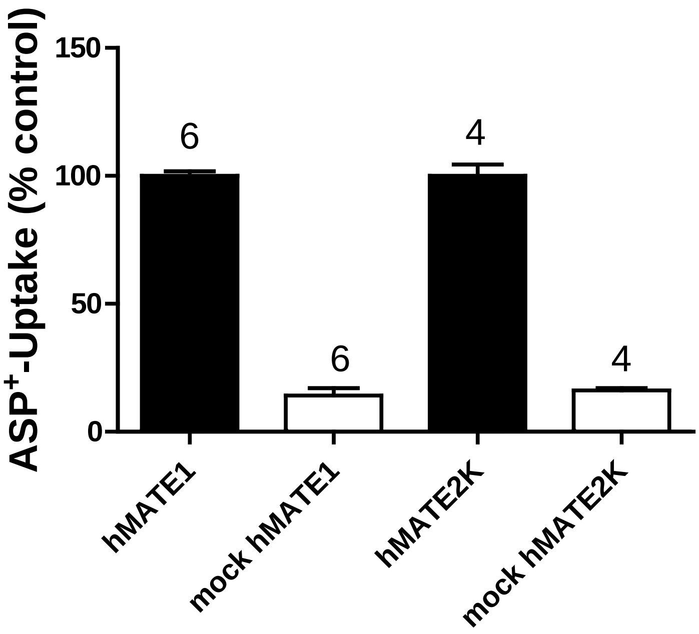

Supplementary Materials Figure 2. ASP^+ uptake measured in hMATE1- (5 µM ASP^+), hMATE2K-(2 µM ASP^+) and the respective mock-transfected cells: The uptake measured in hMATE1 and hMATE2K cells was set to 100%. The mean values ± SEM from 6 and 4 (for hMATE1 and hMATE2K, respectively) independent experiments are shown.

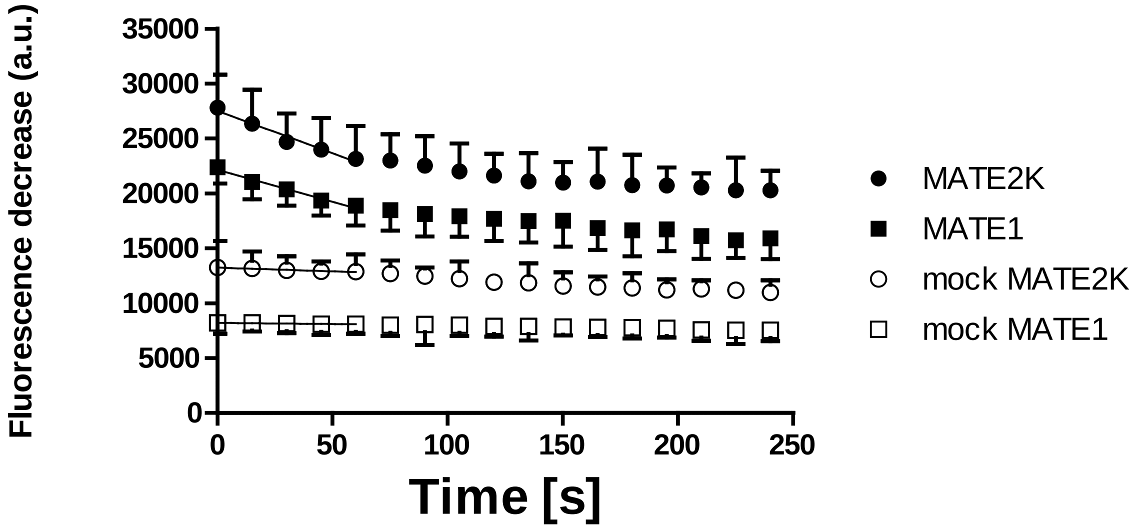

Supplementary Materials Figure 3. ASP^+ efflux measured as fluorescence decrease in arbitrary units (a.u.) in hMATE1-, hMATE2K- and the respective mock-transfected cells: after 10 min incubation with 10 µM ASP^+. The initial fluorescence decrease was much more rapid in hMATE1- and hMATE2K-transfected cells than in mock cells, suggesting that this part of the efflux is mainly mediated by the hMATEs. The mean values ± SEM from 3 independent experiments are shown.

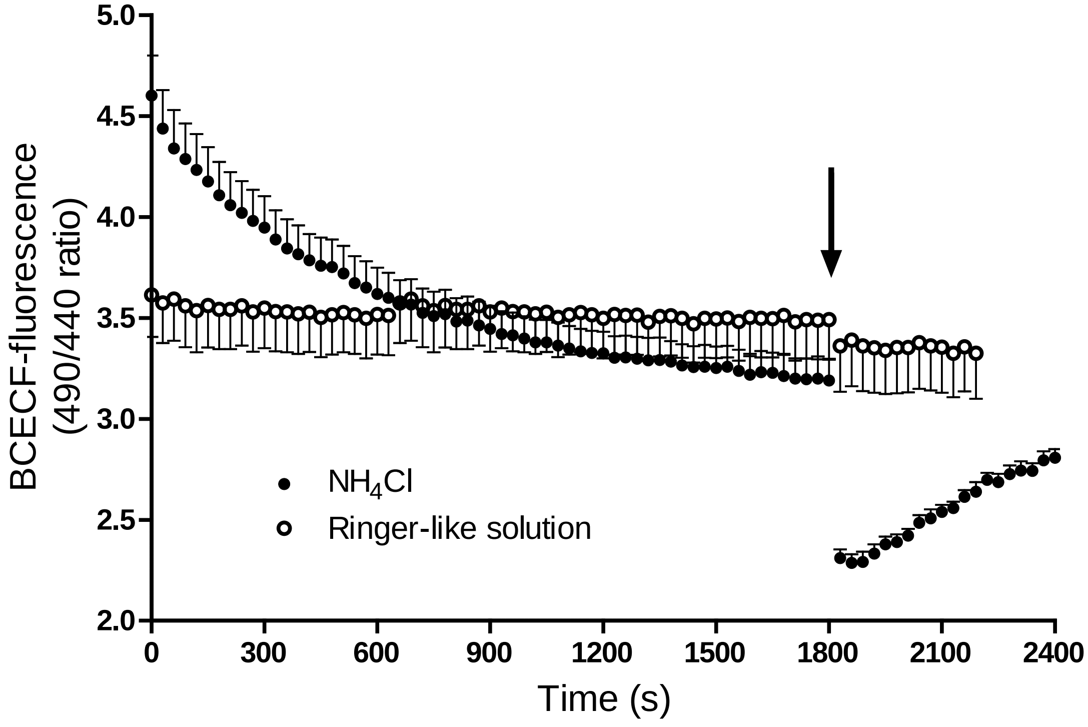

Supplementary Materials Figure 4. Intracellular pH measurement with BCECF-AM represented as ratio of BCECF fluorescent emission measured at 540 nm after excitation at 490 (pH-dependent) and 440 (isosbestic point) nm. After loading with 5 µM BCECF-AM for 30 minutes, cells were incubated with Ringer-like solution (open symbols) or 30 mM NH_4Cl (closed symbols), for 30 minutes. After this, incubation solution was removed and replaced with fresh Ringer-like solution (indicated in the figure with an arrow). The recording of BCECF fluorescence was then continued to show the changes in intracellular pH under the experimental conditions used to study regulation of hMATEs activity. The mean values ± SEM from 3 different experiments with 8-20 replicates are shown.

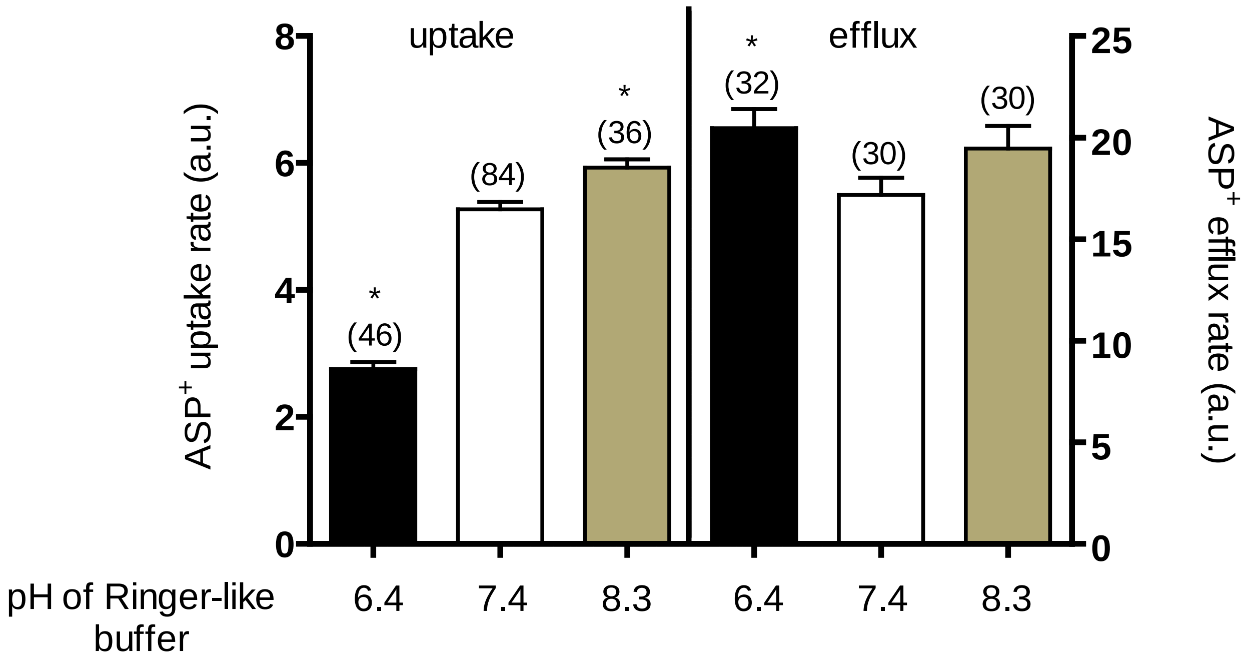

Supplementary Materials Figure 5. Dependency of ASP^+ transport mediated by hMATE1 on the pH of extracellular Ringer-like solution. The uptake of 10 µM ASP^+ showed a clear pH-dependence both in the acidic and basic range. ASP^+ efflux after 30 min loading with 10 µM ASP^+ showed only a clear dependency on the acidic pH. Data are mean ± SEM of several replicates (indicated on the top of the columns) measured in at least 3 independent experiments. * shows a statistically significant difference compared with pH 7.4 ($p<0.05$, Anova with Tukey's multiple comparison test).

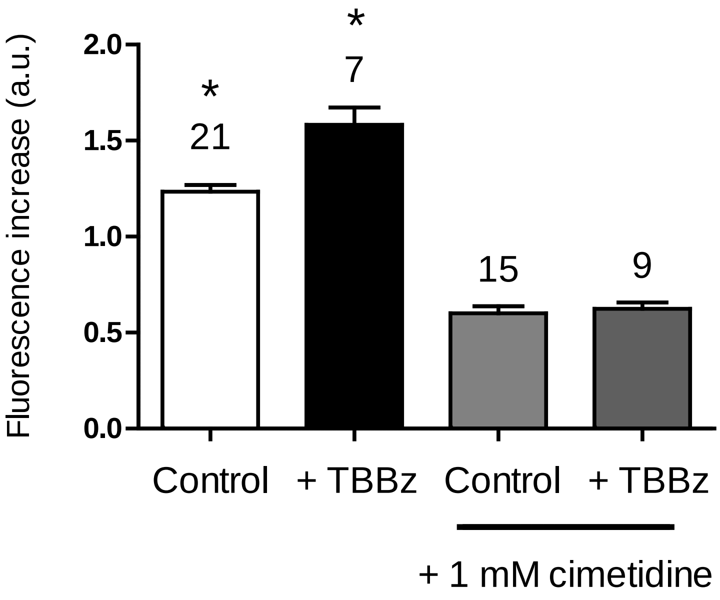

Supplementary Materials Figure 6. Specificity of ASP^+ transport regulation by TBBz in hMATE1-HEK cells. HEK293 cells expressing hMATE1 were incubated with 10 µM TBBz in the presence or not of 1mM cimetidine. The uptake of ASP^+ in wells without TBBz was considered as the control value. The uptake of ASP^+ in the presence of high cimetidine concentration represents the non-hMATE1 ASP^+ uptake and seems to be non-regulable. Data are mean ± SEM of several replicates (indicated on the top of the columns) measured in at least 3 independent experiments. * shows a statistically significant difference compared with all the other values ($p<0.05$, Anova with Tukey's multiple comparison test).

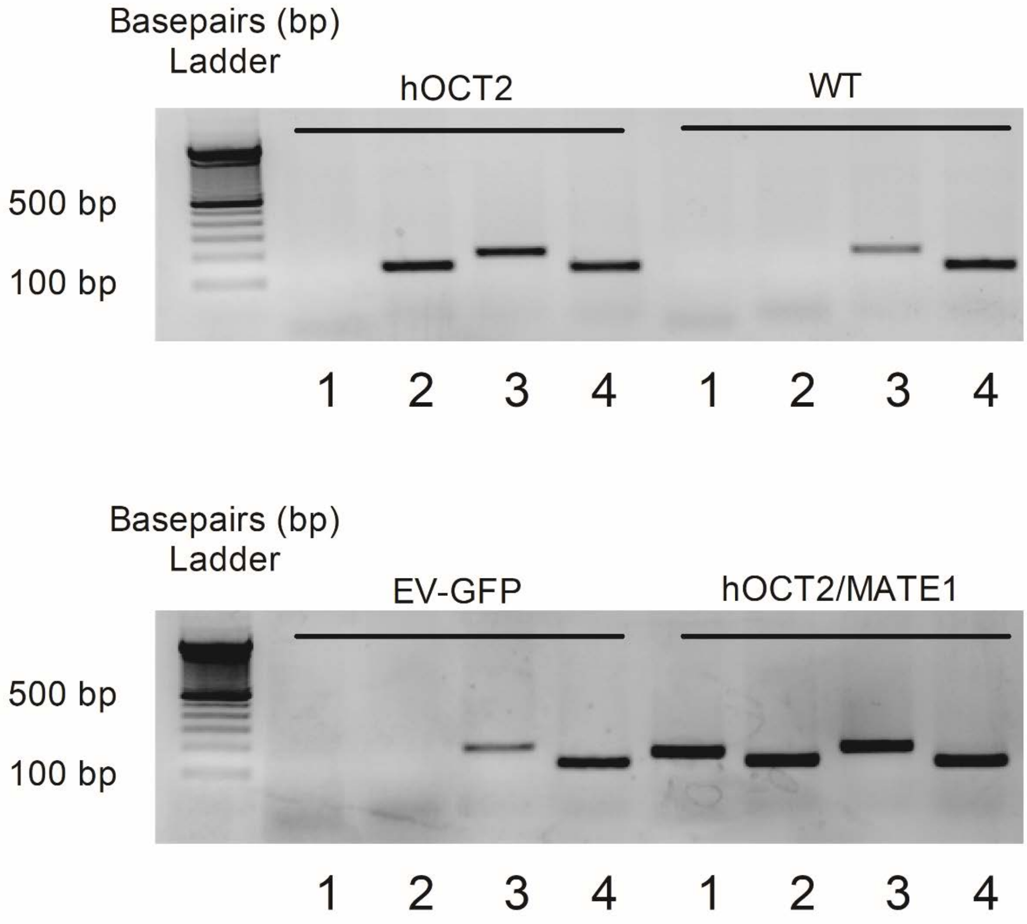

Supplementary Materials Figure 7. RT-PCR analysis of MATE1 and OCT2 expression in MDCK II cells stably expressing hOCT2-GFP alone (hOCT2) or together with MATE1 (hOCT2/MATE1) or stably expressing the empty vector (EV)-GFP or not manipulated (wildtype, WT). 1, 2, 3, and 4 indicate the signals for hMATE1, hOCT2, dog OCT2, and dog GAPDH, respectively. MDCK II cells show an endogenous expression of dog OCT2. Genetic manipulations resulted in hOCT2 and hMATE1 expression.

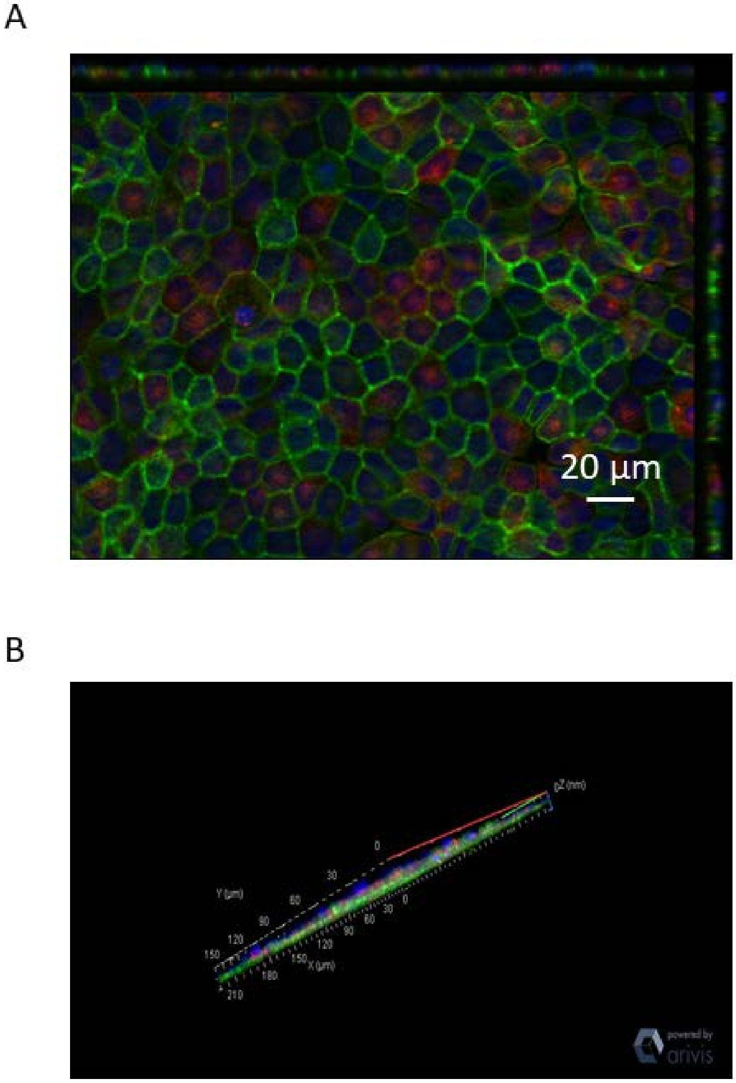

Supplementary Materials Figure 8. Expression of hOCT2 and hMATE1 in MDCK II cells stably expressing hOCT2-GFP after transfection of hMATE1. hOCT2 is well expressed in the basolateral membrane (panel A, green labeling) and hMATE1 (panel A, red labeling) shows a distinct pattern of distribution, mainly associated with the apical membrane, which is more evident in the 3D-reconstitution (panel B) of the stack pictures in panel A using the ZEN software blue edition. In these panels the nuclei are blue-labeled with DAPI.

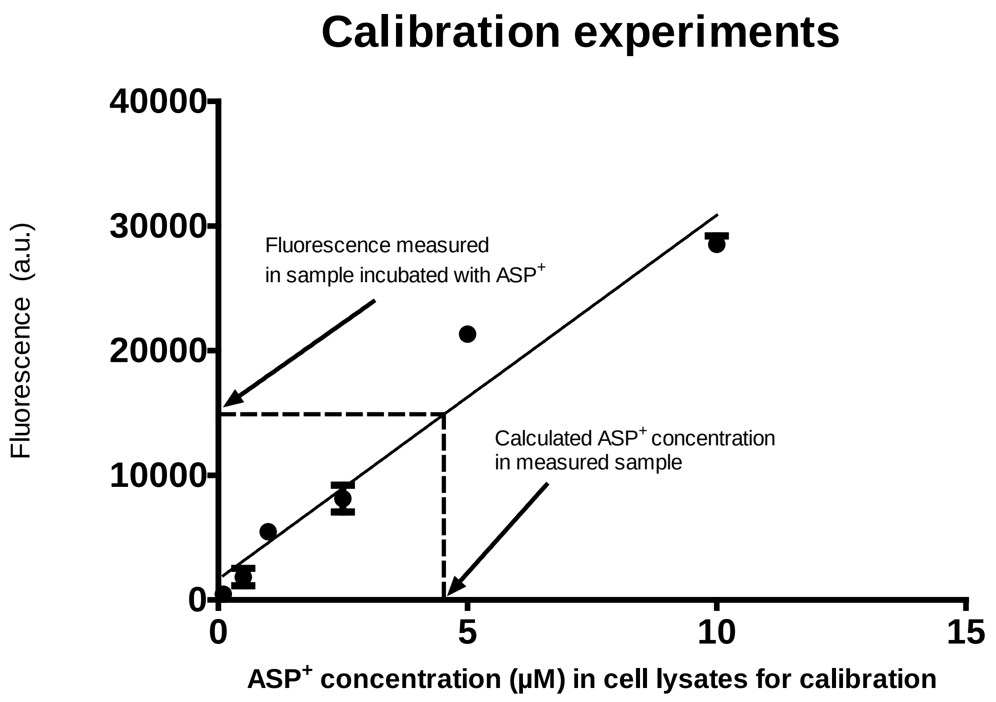

Supplementary Materials Figure 9. Example of calibration experiments for efflux experiments performed in hMATE1-HEK293 cells. Cells used to build the calibration curve were lysed with 4% SDS in 10 mM Tris-HCl and then known amount of ASP^{+} were added to cell lysate. After this, the ASP^{+} associated fluorescence was measured, allowing to calculated the dependency of fluorescence from ASP^{+} concentration. Fluorescence measured in hMATE1-HEK293 cells lysed after incubation with ASP^{+} was used to calculate in an unpaired fashion the intracellular ASP^{+} concentration at the beginning of efflux experiments. The so calculated intracellular ASP^{+} concentration was normalized to cellular protein content.
